# Instrumental and Non-Instrumental Measurements in Patients with Peripheral Vestibular Dysfunctions

**DOI:** 10.3390/s23041994

**Published:** 2023-02-10

**Authors:** Anna Gawronska, Oskar Rosiak, Anna Pajor, Magdalena Janc, Rafal Kotas, Marek Kaminski, Ewa Zamyslowska-Szmytke, Magdalena Jozefowicz-Korczynska

**Affiliations:** 1 Balance Disorders Unit, Department of Otolaryngology, Medical University of Lodz, The Norbert BarlickMemorial Teaching Hospital, 90-153 Lodz, Poland; 2 Department of Otolaryngology, Polish Mother Memorial Hospital Research Institute, 93-338 Lodz, Poland; 3 Audiology and Phoniatrics Clinic, Nofer Institute of Occupational Medicine, 91-348 Lodz, Poland; 4 Department of Microelectronics and Computer Science, Lodz University of Technology, 90-924 Lodz, Poland

**Keywords:** balance disorders, rehabilitation, mobile posturography, clinical tests, wearable devices, smart sensors, medical assistance

## Abstract

Vestibular dysfunction is a disturbance of the body’s balance system. The control of balance and gait has a particular influence on the quality of life. Currently, assessing patients with these problems is mainly subjective. New assessment options using wearables may provide complementary and more objective information. Posturography makes it possible to determine the extent and type of posture dysfunction, which makes it possible to plan and monitor the effectiveness of physical rehabilitation therapy. This study evaluates the effectiveness of non-instrumental clinical tests and the instrumental mobile posturography MediPost device for patients with unilateral vestibular disorders. The study group included 40 patients. A subjective description of the symptoms was evaluated using a questionnaire about the intensity of dizziness using the Dizziness Handicap Inventory (DHI) and Vertigo Syndrome Scale—short form (VSS-sf). The clinical protocol contained clinical tests and MediPost measurements using a Modified Clinical Test of Sensory Interaction on Balance. All patients underwent vestibular rehabilitation therapy (VRT) for four weeks. The non-instrumental measurement results were statistically significant, and the best was in the Timed Up and Go test (TUG). In MediPost, condition 4 was the most valuable. This research demonstrated the possibilities of using an instrumental test (MediPost) as an alternative method to assess balance.

## 1. Introduction

Maintaining balance is the result of the complex integration and coordination of multiple body systems (vestibular, visual, motor, auditory, proprioception), which are centrally processed in the brain [1]. Biomechanically, postural balance is the ability to keep the body’s center of mass (COM) within the base of support with minimal sway [2]. Any impairments of those body systems may cause vestibular disorders.

The economic burden from vestibular disorders is estimated to amount to USD 64,929 across the lifetime of each patient, or a total of USD 227 billion for the population of the USA over the age of 60 [3]. The main causes of increased direct health care costs due to vertigo and dizziness are the many hospital admissions, unnecessarily repeated primary and specialist care consultations, and excessive use of imaging diagnostics. Finally, the patients are often discharged without establishing the etiology, and, therefore, they are not prescribed the appropriate therapy [4]. Moreover, the indirect costs due to vertigo and dizziness are noted. There is a lack of autonomy, a fear of falls, and related changes due to the forced sedentary lifestyle. A reduced capacity to work or needing assistance in activities of daily living is observed.

The rehabilitation of balance dysfunction is based on exercises, which stimulate compensation and habituation processes on different levels [5]. Hall et al. recommended vestibular rehabilitation for peripheral vestibular hypofunction in the Clinical Practice Guideline of the American Physical Therapy Association (APTA) [6]. Early and active vestibular rehabilitation therapy (VRT) is essential to achieve compensation. The results should be confirmed by the patient’s subjective self-assessment, such as questionnaires and objective measurements. The quantitative measure of the severity of the disease can be assessed using questionnaires such as the Dizziness Handicap Inventory (DHI) or Vertigo Symptom Scale (VSS), which attempt to evaluate the multifactorial nature of these disorders [7,8].

The effectiveness of rehabilitation can be assessed with clinical tests, the most popular of which are the Timed “Up and Go” test (TUG), the Dynamic Gait Index (DGI), the Berg Balance Scale (BBS), the Tinetti test (TT), and the Functional Reach test (FR) [9,10,11,12]. Clinical balance tests usually consist of static and dynamic tasks, while some even attempt to estimate the risk of falls. To complete the examination, the score is measured in points or time, which is then stratified into different subpopulations according to the risk of a fall. 

The use of non-instrumental measurements has certain advantages, including that they are easy to interpret, do not require expensive equipment, and are easy to perform in an outpatient setting. The disadvantages are that some of the tests focus only on categorizing the risk of falling or on gait assessment, while others focus on static balance. To achieve a comprehensive evaluation of a dizzy patient, several tests are necessary, which is time-consuming. There are some difficulties in selecting a complete balance assessment tool that would meet all a clinician’s expectations. Moreover, some authors have stated that clinical tests are not sensitive enough to detect subtle changes, either worsening or improving the patient’s balance abilities [2,13]. The evaluation of non-instrumental tests can be subjective and might be biased by the expectations of the physician or patient regarding improvement after therapy, so objective measurements are needed.

Static posturography, also called stabilometry, has been used for over 35 years and is performed to assess only static conditions. The subject stands on a fixed or tilted platform on a fixed support base. There are some variants of stabilometry, such as the one- or two-foot stance, firm or foam surface, or open and closed eyes. The other type of posturography is computerized dynamic posturography (CDP), wherein a force platform has been combined with visual stimuli as a means of determining the relative importance of the various sensory inputs critical for balance, namely, vision, somatosensation, and vestibular sensation. CDP detects postural sway by measuring shifts in the center of gravity (COG) as a person moves within their limits of stability [14]. Objective measurement of static posturography can be done with inertial sensors [15,16]. The development of modern mobile posturography based on wearable sensors provides the means to register small, sensitive changes in the functioning of postural control [13]. Currently, there are different commercial systems that use mobile devices to diagnose and rehabilitate balance disorders or detect falls in the elderly [17,18]. These devices have also found a role in the treatment of various neurological diseases, such as Parkinson’s disease, multiple sclerosis, or Alzheimer’s disease. However, there is a lack of standardization in data acquisition, mathematical models, and algorithms used to process the data.

Balance impairments are one of the leading causes of falls. The consequences of falls may be directly linked to an increase in mortality—such as a lower limb or pelvis fracture. Furthermore, suffering a fall can also cause “post-fall” syndrome, a psychomotor regression condition responsible for psychological, postural, and gait dysfunction, mainly in elderly people. Some clinical tests used in this study (TUG, DGI, Tinetti) assessed the risk of falls. However, clinical assessment is subjective and is not sensitive enough to identify early balance dysfunction. Instrumental measurements may make it possible to detect early subclinical postural changes in daily conditions. Wearable devices can be used for long-term monitoring for preventive and recovery strategies; thus, individualized strategies for fall prevention could be created (e.g., the use of mobility aids, changing environmental hazards, or rescue interventions) [19].

The aim of the study was to evaluate the usefulness of non-instrumented and instrumented measurements in patients with peripheral vestibular dysfunctions. 

## 2. Materials and Methods

The study included 40 patients, 20 women and 20 men with a mean age of 56.8 ± 14 years old, complaining of vertigo and balance disequilibrium, who were diagnosed at the Balance Disorders Unit, Otolaryngology Department, Medical University of Lodz. 

The inclusion criterion was a lack of spontaneous compensation within one month after unilateral peripheral impairment, which was confirmed using the results of videonystagmography (VNG).

Exclusion criteria were central vestibular signs in VNG, bilateral peripheral vestibular loss, disorders of the motor system, and coexisting neurologic disorders. 

Patients were interviewed for history related to balance dysfunction and coexisting diseases according to the self-assessment survey and the questionnaire about the intensity of dizziness using the DHI and the Vertigo Symptom Scale—short form (VSS-sf). The DHI is a 25-item self-report questionnaire that assesses the impact of dizziness and balance dysfunction on the quality of life. The maximum score is 100 points (severe, moderate, and mild handicap with 61–100, 31–60, and 0–30 points, respectively) and a minimum score of 0. There are three subscales: Physical (P), Functional (F), and Emotional (E). Although it was originally written in English, DHI has been translated into many languages, e.g., Polish, German, Norwegian, Brazilian, and Spanish [20]. The other common, widely used questionnaire is the Vertigo Symptom Scale, published by Yardley in 1992. The objective of this scale is to measure the frequency of balance and vertigo symptoms and autonomic/anxiety symptoms [21]. Currently, a short form of VSS (VSS-sf) is used. The 15-item VSS-sf is divided into 2 subscales: vertigo–balance (VSS-V), which refers to vestibular symptoms, and autonomic–anxiety (VSS-A). A general result of ≥12 points means severe dizziness/vertigo [22]. 

All patients underwent otoneurologic examination, including five clinical tests: the Timed Up and Go test, the Dynamic Gait Index, the Berg Balance Scale, the Tinetti test, and the Functional Reach test. The characteristics of the tests are presented in Table 1.

VNG examination (Ulmer SYNAPSIS 2008) was performed, including a caloric test, kinetic stimulation with torsion swing, and positional and oculomotor tests (saccadic, smooth pursuit, optokinetic). Peripheral unilateral vestibular impairment was diagnosed when there was asymmetry of the vestibular response in a bithermal water caloric test (44° and 30 °C by Fitzgerald-Hallpike) and canal paresis (CP) was >22%. Central vestibular signs in VNG were diagnosed when there were abnormalities in saccades (prolonged latency, hyper or hypometrics), smooth pursuit (low gain, morphology), or an optokinetic test (low gain) on incorrect morphology recordings [28]. 

Postural stability was measured using the portable, battery-powered MediPost system with one sensor mounted on the trunk at the L5 level [16]. Mobile posturography allows for a more direct measurement of COM, which is strongly correlated with the center of pressure (COP) [29]. The system consisted of the ESP32 system, a Wi-Fi radio module, and the tri-axis inertial measurement unit (IMU; STMicroelectronics LSM9DS1) based on a microelectromechanical system (MEMS) that included an accelerometer, a gyroscope, and a magnetometer (manufactured by University of Technology, Lodz, Poland). This kind of IMU is particularly suitable for measuring low angular speeds (low sway frequencies). The device was synchronized and controlled using a computer program via a Wi-Fi network. A sampling frequency of 200 Hz is used on the IMU device. Then a low-pass filter is also implemented on the IMU, after which the signal is represented with 20 samples per second. The samples are sent after the measurement is completed. The following step is used to determine the device’s angular position using the Madgwick approach [30]. A detailed description of the system can be found in our previous publication [16].

Determining the angular position of the MediPost device allows for the computation of the following measures that were used for further analysis: the total length of trajectory, which is the length marked by a projection of the patient’s center of pressure excursion (LEN—mm), stabilogram expanded area (SURF—mm^2^), maximum angular velocity (MAXAV—deg/s), and mean angular velocity (MEANAV—deg/s).

**Figure 1:**
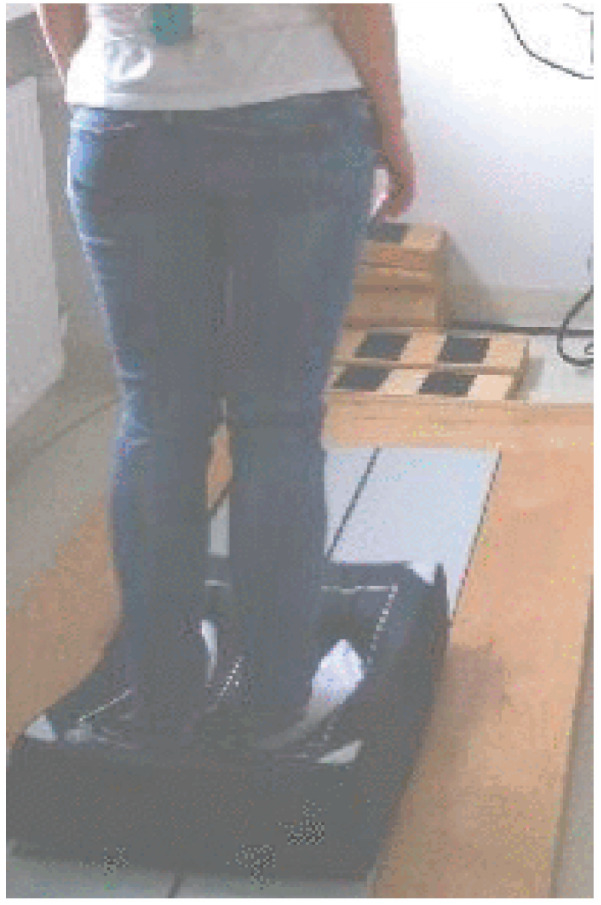
The posture in the posturographic test.

Four posturographic tests were performed: standing on a firm (condition 1) and foam (condition 3) surface with eyes open and standing on a firm (condition 2) and foam (condition 4) surface with eyes closed. The subjects were asked to stand with their hands at their sides, feet apart, on a surface with rectangular boundaries of 45 × 45 cm (foam 45 × 45 × 12.3 cm) (Figure 1).

All patients underwent a rehabilitation program for four weeks, and each session lasted 60 min. The rehabilitation program was supervised by a physiotherapist [31]. In accordance with APTA guidelines, they performed VRT based on Cawthorne–Cooksey exercises, which involved improving posture coordination and spatial orientation, as well as optokinetic training [32]. One particular vestibular exercise included augmented sensory feedback, and the target was to identify activity limitations and the patient’s restrictions.

All participants were fully informed about the aim of the study and the test procedure, and they gave informed consent. The study design was approved by the Ethics Committee of the Medical University of Lodz (RNN/136/16/KE, 10 May 2016).

## 3. Results

### 3.1. Statistical Analysis

Statistical analysis was performed using the R Project for Statistical Computing (ver. 3.6.3). The data were expressed as means ± standard deviation (SD) and checked for normality with the Shapiro–Wilk test. The paired Student’s *t*-test was used for the questionnaires and clinical test data, and log-transformed data of the mobile posturography was used to compare the groups. The differences were considered significant at *p*-value < 0.05. The relative difference between the pre-VRT and post-VRT measures was correlated using Pearson’s rank correlation.

### 3.2. Results of the Non-Instrumental Measurements 

#### 3.2.1. Questionnaires 

The total result of the DHI questionnaire was 53.9 points before VRT, which decreased by 33% to 36.3 points after VRT (*p* < 0.001). The result is statistically significant (*p* < 0.001). Improvement was visible in all subscales of the DHI, with the greatest change in the emotional subscale: 17.3 before VRT vs. 10.4 after, which is a 40% improvement.

In VSS-sf, the mean score for the whole group at the initial examination was 19.7, which decreased significantly to 11.9 points (*p* < 0.001) after VRT, a 40% reduction in the subjective intensity of symptoms. The results of the VSS-sf confirmed the improvement in the patient’s physical perception of vertigo in the VSS balance subscale, as well as the emotional burden related to vertigo in the anxiety subscale. The difference was greater in the balance subscale by 43%, which is related more to the physical perception of vertigo (Table 2). 

#### 3.2.2. Clinical Tests 

The clinical tests revealed a statistically significant improvement in postural stability in all functional trials (Table 3). The greatest change was noted in TUG, by 31%, and DGI, by 13% (12.4 vs. 8.5 s, respectively, *p* < 0.001, and a mean score of 53.9 vs. 36.8 points, respectively; *p* < 0.001). In the initial TUG test, 45% had a result of >12 s, which is interpreted as a high fall risk. After VRT, this group decreased to only 5% of patients at the final evaluation. The total time to complete TUG after intervention improved by almost 4 s (mean 12.4 vs. 8.5, respectively, *p* < 0.001). Before VRT, 55% of the subjects were categorized as likely fallers, with a DGI result of ≤19 points, while after VRT, only 22.5% were in that category. Significant differences were also found in BBS and TT (mean score of 49.9 vs. 52.5, *p* < 0.001, and 29.2 vs. 32.8 points, *p* < 0.001, respectively). The percentage improvement for those tests was 5% for BBS and 10% for TT. In the FR test, the average results improved by 3 cm, which is 12% more than at the initial evaluation. The fall risk decreased by half in 17.5% of subjects who reached more than 24 cm (Table 2).

**Figure 2:**
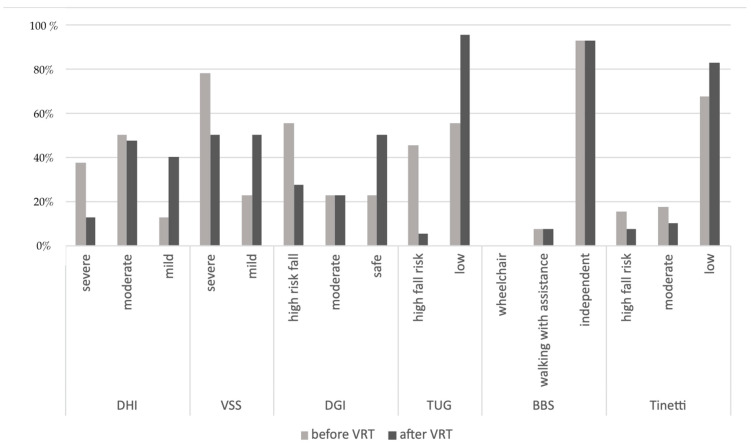
The non-instrumental tests—the percentage distributions of patients before and after VRT.

Figure 2 presents the percentage distributions of patients in the non-instrumental tests. In the DHI questionnaire, almost 88% of patients before VRT were classified as severe and moderate handicap, whereas after VRT, mild handicap was noted in 40%. Initially, almost 78% of the group was classified as severe on the VSS-sf scale, while after treatment, 50% was in the mild category. The DGI test can divide patients into high and moderate risk of falls, which was 55% and 22.5% of the subjects before VRT, respectively; however, after therapy, 50% of patients fell into the no fall risk category. A high fall risk was noted in 45% of patients in TUG; however, after VRT, it changed to 95% of patients in the low fall risk category. The BBS test categorized the population into three groups: wheelchair-bound, walking with assistance, and independent. In this study, one of the exclusion criteria was a motor disorder, which influenced the distribution of patients in this test by eliminating patients in the wheelchair group; the distribution of the BBS did not change after therapy. Initially, in the TT test, 15% and 17.5% presented a high and moderate fall risk, respectively; after VRT, 82.5% had a low risk of falls. 

### 3.3. Results of the Instrumental Measurements

#### MediPost Posturography 

Four posturographic measures were selected for analysis: the length, surface, maximum angular velocity, and mean angular velocity of COP displacement in time. The greatest differences between evaluations were observed in sensory condition 4, the most difficult—eyes closed on foam. Those results were statistically significant at *p* < 0.01 in all analyzed measures. A decrease in all measures was also observed in condition 3, except for maximum angular velocity, where there were no differences after the intervention. Similarly, no differences among all analyzed measures were observed in the least sensory-challenging trial—condition 1, eyes open on a stable surface (Table 3). 

### 3.4. Correlation of Improvement between Instrumental and Non-Instrumental Measurements

**Figure 3:**
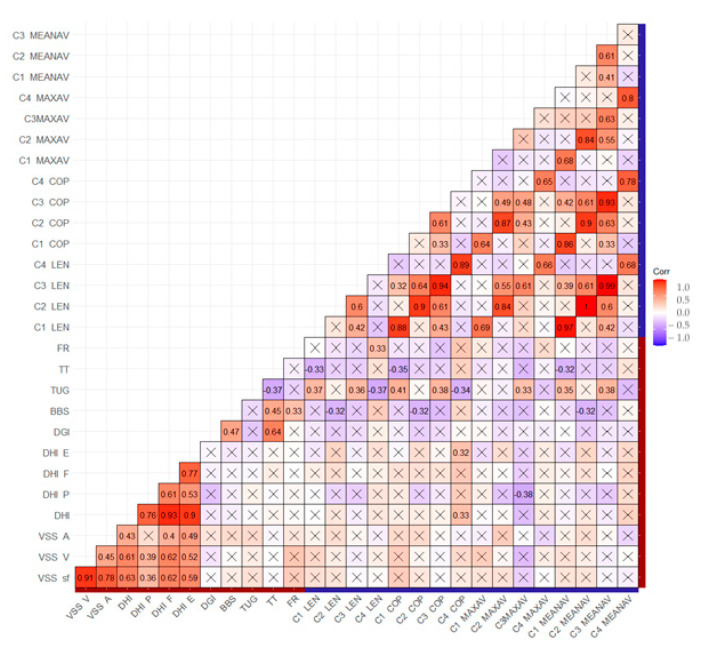
Correlation matrix of improvement between instrumental and non-instrumental measurements. The red boxes apply to Pearson’s correlation coefficient r = 1.0, and the blue boxes to r = −1.0. A lighter color indicates a correlation close to 0 or r = 0 (white). The X-marked boxes: non-significant values. The red line in the axes: non-instrumental measures; the blue line in the axes: instrumental measures. C1—eyes open, firm surface, C2—eyes closed, firm surface, C3—eyes open, foam surface, C4—eyes closed, foam surface. LEN—length of trajectory, COP—surface of COP, MAXAV—max angular velocity, MEANAV—mean angular velocity.

The results of the correlation between the questionnaires, clinical scales, and objective measurement are presented in the matrix (Figure 3). A clinically significant correlation was observed between the TUG test and MediPost in condition 3 for all measures, conditions 1 and 4 for the length of trajectory and surface of COP, and condition 1 for mean angular velocity (*p* < 0.01, respectively).

A moderate positive association between before-VRT mobile posturography for all measures for condition 2 and the after-VRT outcomes of VSS and a moderate association between conditions 3–4 and after-VRT outcomes of the DHI questionnaire were noted. Negative associations can be observed between conditions 1 and 3 in terms of the length of trajectory, the surface of COP, maximum and mean angular velocity, and the after-VRT outcomes of DGI, BBS, and the strongest association with the Tinnetti after VRT scoring. This correlation matrix is presented in the Supplementary Material.

## 4. Discussion

This study revealed the results of non-instrumental clinical tests and instrumental measurements achieved using the novel MediPost mobile device. Mobile posturography evaluation shows an improvement in postural stability in the study population, with differences depending on the test conditions. Those results are useful in assessing the effectiveness of vestibular rehabilitation in patients with peripheral vestibular dysfunction.

Our group of patients suffered from vertigo, dizziness, and balance unsteadiness due to the lack of spontaneous compensation after unilateral peripheral impairment. The patients’ stability was evaluated twice—before rehabilitation and one month after, in the same way, using non-instrumental (clinical tests) and instrumental tools (mobile posturography). A complete evaluation of patients with instability should include a psychological aspect of the disease, which was checked in this study using DHI and VSS-sf.

Based on a few high-quality randomized controlled trials, moderate to strong evidence exists that vestibular rehabilitation is safe, effective management for unilateral peripheral vestibular dysfunction [31,33], and the results of this study are in line with this statement. Multiple studies confirmed improvements in postural stability after VRT [6,34,35]. In patients with vertigo and balance disorders, Brown et al. demonstrated a reduction in the overall DHI score and shortened TUG time after VRT, which was also observed in our study [36].

We conducted, a few clinical tests to investigate many aspects of stability, such as static and dynamic balance, assessment of gait, and fall risk. The TUG and DGI tests evaluate dynamic balance and gait quality, while TT and BBS focus on static and dynamic tasks [2]. The FR test assesses only one condition of dynamic stability—the anterior displacement within the limits of stability. Among all the aspects of balance that were evaluated, we noted the greatest improvement in dynamic tasks in the TUG test by 31%. This test is less complicated than DGI, which influenced the result. Initially, the group of patients obtained quite good results in BBS and TT, and for this reason, the improvement here was not as visible as in the tests with dynamic components; however, the results are still statistically significant. Many studies stated that the improvement in clinical tests means a lower risk of falls and, thus, better postural stability [32,35,37,38]. 

Patients with peripheral vestibular deficits often show instability during quiet stance tasks. It is well established that removing visual inputs by closing the eyes or reducing the efficacy of lower-leg proprioceptive inputs by destabilizing the support surface, e.g., by using foam [39], increases the sensitivity of quiet stance trials toward detecting vestibular deficiencies. Lacour et al. also stated that vestibular inputs are more crucial to keep balance in more challenging postural tasks on unstable supports with eyes closed or with moving surroundings [40]. 

The instrumental measurement performed using MediPost mobile posturography localized at the L5 level, assessed postural stability in different conditions where the noted grade of improvement varied, as stated in the literature [13,39,40,41]. The results of condition 4 (standing on foam, eyes closed) were statistically significant for all MediPost measures, which is consistent with other studies [5,29,42]. The results of condition 1 (firm surface, eyes open) and condition 2 (firm surface, eyes closed) are clinically unsatisfactory.

In the literature, recovery in undemanding balance conditions was generally seen over weeks and months. However, recovery of dynamic postural function took more time, and compensation was incomplete in more challenging postural conditions [13,40]. One of the inclusion criteria in this study was a lack of spontaneous compensation within one month after unilateral peripheral impairment. During this period, spontaneous compensation could occur to some extent, as is seen in the results of less challenging conditions 1 and 2. Basta et al. analyzed mobile posturography of daily life mobility and concluded that proprioceptive input had a greater impact on postural control than visual input during the two-leg stance tasks for all age groups [41]. 

The main goal of VRT is to restore or improve dynamic functions [5]. Weight shifting in stance is used to improve center of gravity control and balance recovery [31]. Balance with eyes closed and when somatosensory input is altered by standing on foam invokes changes in the base of support. Those conclusions were also observed in this study, where in the instrumental measurements, the “more difficult” conditions 3 and 4 could be used to diagnose vestibular dysfunction. Allum et al. noted that only two of the four two-legged stance tasks that are usually performed (with eyes closed both for normal and foam support) are worth recording for balance screening [39]. 

Our study analyzed the relationship between clinical scales and objective measurement. The only significant correlation was noted between the TUG test and Medipost in several conditions and for some measures. By contrast, O’Sullivan et al. observed a correlation between BBS, TUG, and accelerometry [38]. The mean scores of non-instrumental measurements of this study population also represent a relatively high-functioning group, and the ability to maintain balance between eyes open and eyes closed should be well within their control. Izquierdo et al. did not find a correlation between the DHI and static balance measurement; however, a greater correlation was noted in SwayStar, which could show that dynamic balance is perceived as more disabling for the study group.

The remaining analysis generally shows no clinically significant correlations between instrumental and non-instrumental measurements, even though an improvement is noted in both categories, which was also concluded in other publications [43,44,45,46]. Mbongo et al. did not observe correlations between DHI and dynamic posturography in patients with unilateral vestibular loss [44]. Yip and Strupp did not find correlations between the DHI and caloric tests, cervical/ocular vestibular-evoked myogenic potentials, and posturographic measures [45]. The lack of correlation between the above-mentioned methods may be explained by a subjective bias in clinical scales performed by the physician and the self-performed scales by the patient, insensitivity to mild impairments (ceiling effects), and poor reliability. Objective measures are free of such bias. Furthermore, the clinical tests are related in that they all assess various aspects of balance and mobility, but they have different domains of balance, such as the risk of falls, gait performance, and dynamic aspects of balance. Therefore, although an improvement may be noted, a correlation might not be observed. In the literature, it is stated that the influence on the correlation of those measure methods has unaccounted factors mostly at the socio-behavioral level [34,45]. Both non-instrumental and instrumental measures have their own strengths and limitations, and it is important to use a combination of both types of measures to gain a comprehensive understanding of an individual’s balance dysfunction.

The cost and size of mobile devices make them easy to use at home for continuous monitoring and home rehabilitation, which, as a result, opens several valuable prospects for clinicians in telemedicine and telerehabilitation. The low-cost tools for VRT monitoring and screening compared to classic static or dynamic posturography can make this a more available method. In the future, the technological migration of wearable sensors from the laboratory to the domestic environment is needed [47]. The wearable nature of such systems may open the way to assess balance not only in static conditions but also during dynamic daily activities where normal posturography cannot be used.

## 5. Conclusions

Clinical tests and posturographic measurements using the mobile MediPost system provide an assessment of patients with peripheral vestibular dysfunctions. This research demonstrated the possibilities of using an instrumental test (MediPost) as an alternative method to evaluate balance deficits. Ongoing development and testing of inertial sensors are necessary before employing the technology as a replacement for current clinical tests. There are some limitations in this study. The study group was homogenous and involved only patients with unilateral vestibular dysfunction. Furthermore, we performed instrumental measurements only in a quiet stance. Further research should include larger populations of patients with balance problems with age-matched controls and more dynamic tests.

## Figures and Tables

**Table 1:** Clinical balance tests.

Clinical Test	Purpose	Number of Tasks	Score	Total	Interpretation	References
**The Timed Up and Go Test (TUG)**	Dynamic balance, fall risk	1	Time (seconds)	-	>12 s *	[2,23]
**Dynamic Gait Index (DGI)**	Dynamic balance	8	0–3 (points)	24	≤19/24 predictive of falls in the elderly >22/24 safe ambulators	[2,24]
**Berg Balance Scale (BBS)**	Static and dynamic balance	14	0–4 (points)	56	0–20—wheelchair-bound, 21–40—walking with assistance, 41–56, independent	[2,25]
**The Tinetti test**	Static and dynamic balance	16 (9 balance-, 7 gait-related)	0–1; 0–2 (points)	28	fall risk ≤ 18—high; 19–23—moderate; ≥24—low	[26]
**The Functional Reach test (FR)**	Dynamic balance	1	centimeters	-	≥25 cm—low risk of falls, 15–24 cm—risk of falling is 2× greater	[2,27]

* Recommended value; there are other cut-offs, dependent on the examined group.

**Table 2:** Results of non-instrumental measurements.

Non-Instrumental Test		Before VRT	After VRT	MD	***p*-Value**
**DHI (points)**	**Total**	53.9 ± 18.7	36.3 ± 20.6	−17.7	***
	**Physical**	15.5 ± 7	11.3 ± 6.9	−4.2	***
	**Functional**	21.2 ± 7.8	16.2 ± 9.7	−5	**
	**Emotional**	17.3 ± 7.9	10.4 ± 7.8	−7	***
**VSS-sf (points)**	**Total**	19.7 ± 9.3	6.9 ± 5.1	−7.9	***
	**Vertigo–balance**	12.1 ± 6	6.9 ± 5.1	−5.2	***
	**Autonomic–anxiety**	7.7 ± 5.3	5.0 ± 4.2	−2.7	***
**TUG (seconds)**		12.4 ± 5	8.5 ± 2.5	−3.9	**
**DGI (points)**		18.7 ± 4.1	21.1 ± 3.9	2.4	**
**BBS (points)**		49.9 ± 5.4	52.5 ± 5.5	2.6	**
**Tinetti (points)**		23.7 ± 4.5	26.0 ± 3.1	2.3	**
**FR (points)**		29.2 ± 8.4	32.8 ± 8.4	3.6	*

Before VRT mean ± SD; After VRT mean ± SD; MD—mean difference; The paired Student’s *t*-test ***—significant at the <0.001 level, **—significant at the <0.01 level, *—significant at the <0.05 level, ns—no statistical significance.

**Table 3:** Results of instrumental measurements.

Posturography Condition	Before VRT	After VRT	Before VRT	After VRT	Before VRT	After VRT	Before VRT	After VRT
	Posturography Measure							
	Length of Trajectory (mm)		Surface of COP (mm^2^)		Max Angular Velocity (deg/s)		Mean Angular Velocity (deg/s)	
**Condition 1**	86.7 ± 45.2	73 ^ns^ ± 23.2	151.5 ± 208.6	92.1 ^ns^ ± 69.6	2.8 ± 1.2	2.6 * ± 1.3	0.5 ± 0.3	0.4 ^ns^ ± 0.1
**Condition 2**	143.7 ± 146.6	114.5 ^ns^ ± 82	508.8 ± 1435.1	198.5 ^ns^ ± 245.1	3.6 ± 2.7	3.3 ^ns^ ± 1.5	0.8 ± 0.9	0.7 ^ns^ ± 0.5
**Condition 3**	213.5 ± 130.7	169.5 ** ± 82.5	1118.6 ± 1712.5	759.1 ** ± 1237.7	4.5 ± 3.1	3.7 ^ns^ ± 1.5	1.3 ± 0.9	1 ** ± 0.5
**Condition 4**	373.9 ± 172.7	301.7 ** ± 122.6	3931.2 ± 5577.5	2381.6 ** ± 2055.8	7.8 ± 4	6.1 *** ± 3	2.6 ± 1.4	2 *** ± 0.9

Before VRT mean ± SD; After VRT mean ± SD; condition 1: eyes open, firm surface; condition 2: eyes closed, firm surface; condition 3: eyes open, foam surface; condition 4: eyes closed, foam surface. The paired Student’s *t*-test for logarithmically-transformed data ***—significant at the <0.001 level, **—significant at the <0.01 level, *—significant at the <0.05 level, ns—no statistical significance.

## Data Availability

The data presented in this study are available on request from the corresponding author. The data are not publicly available due to restrictions imposed by the funding institution.

## Supplementary Materials

The following supporting information can be downloaded at: https://www.mdpi.com/article/10.3390/s23041994/s1.
