# Supplementary material for: Instrumental and Non-Instrumental Measurements in Patients with Peripheral Vestibular Dysfunctions
Source: Sensors (Basel). 2023 Feb 10;23(4):1994. doi: 10.3390/s23041994 (PMC9963841; doi:10.3390/s23041994)
Supplement: Supplementary file 1 [file sensors-23-01994-s001.zip › sensors-2169590-supplementary.pdf]

Color correlation matrix

N=40

r>=	-1	-0,80	-0,60	-0,40	-0,20	0	0,20	0,40	0,60	0,80	1

Variable	LEN-O 2	LEN-C 2	LEN-O FOAM 2	LEN-C FOAM 2	SURF-O 2	SURF-C 2	SURF-O FOAM 2	SURF-C FOAM 2	MAXAV-O 2	MAXAV-C 2	MAXAV-O FOAM 2	MAXAV-C FOAM 2	MeanAV-O 2	MeanAV-C 2	Mean-O FOAM 2	Mean-C FOAM 2	VSS 2	DHI 2	DGI 2	BBS 2	Tinetti 2	FR 2	TUG 2
LEN-O	0,605904	0,530281	0,222643	0,167033	0,636997	0,588842	0,093854	0,364799	0,087100	0,284435	-0,005450	0,336300	0,590832	0,535679	0,184090	0,260402	0,283429	0,210311	-0,477627	-0,393043	-0,642105	-0,142589	0,226320
LEN-C	0,395527	0,536003	0,156090	0,329004	0,429582	0,600795	0,157938	0,495138	0,187423	0,321211	0,046090	0,461095	0,370731	0,528353	0,153181	0,407035	0,421088	0,241635	-0,254390	-0,282879	-0,573729	-0,033362	0,027379
LEN-O FOAM	0,533669	0,461301	0,573185	0,296505	0,488290	0,562462	0,499544	0,594761	0,138645	0,252029	0,358472	0,571611	0,500151	0,452830	0,560683	0,585742	0,244817	0,315668	-0,551500	-0,529263	-0,776839	-0,314872	0,182782
LEN-C-FOAM	-0,048194	0,148037	0,127563	0,309542	-0,059170	0,059879	0,190500	0,145863	-0,096745	0,233102	0,081150	0,200379	-0,054398	0,141239	0,142140	0,289840	-0,065677	0,033754	0,069290	0,060952	0,093057	-0,070935	0,143950
SURF-O	0,499140	0,464108	0,129113	0,141723	0,599220	0,618633	0,022697	0,412282	0,159543	0,284753	-0,002410	0,302745	0,500025	0,475088	0,100653	0,230443	0,274510	0,200940	-0,452807	-0,319081	-0,598793	-0,108968	0,202140
SURF-C	0,298373	0,366812	0,053478	0,263364	0,366760	0,520968	0,021649	0,515574	0,209578	0,231299	0,050404	0,438422	0,289528	0,367740	0,048451	0,349261	0,353940	0,220514	-0,292520	-0,255716	-0,565708	-0,035929	0,014440
SURF-O FOAM	0,442038	0,428690	0,443469	0,215758	0,423860	0,595220	0,435620	0,556001	0,146790	0,239603	0,334194	0,508288	0,417039	0,421269	0,443647	0,481189	0,209780	0,250640	-0,435387	-0,376389	-0,688230	-0,188209	0,100644
SURF-C FOAM	-0,111463	0,077651	-0,031565	0,152374	-0,051664	0,025069	0,020429	0,076350	-0,209821	0,228961	0,027475	0,145967	-0,116017	0,075718	-0,021037	0,172781	-0,218681	-0,158987	0,022978	0,048005	0,054061	-0,123359	0,188771
MAXAV-O	0,162602	0,213938	-0,093318	0,015799	0,259509	0,315460	-0,146365	0,164039	-0,076402	0,123844	-0,010542	0,171665	0,207914	0,239318	-0,101770	0,119862	0,147550	0,191309	-0,182800	-0,026929	-0,198424	0,021991	0,112580
MAXAV-C	0,297330	0,392157	0,021870	0,353620	0,318797	0,509774	0,020628	0,519107	0,268463	0,289498	-0,032030	0,484274	0,290585	0,390707	0,023010	0,400998	0,401489	0,215697	-0,166690	-0,171082	-0,460581	0,040894	-0,030760
MAXO-FOAM	0,584914	0,497983	0,482537	0,142944	0,569510	0,636832	0,504160	0,480033	0,206543	0,272022	0,261992	0,426953	0,562610	0,494227	0,505512	0,459709	0,301210	0,366504	-0,478928	-0,420981	-0,690238	-0,250681	0,184371
MAX-C-FOAM	0,150389	0,344294	0,192430	0,389394	0,133184	0,347535	0,220345	0,454507	-0,091798	0,370678	0,117088	0,522470	0,155937	0,344825	0,209543	0,521710	0,104070	0,202563	-0,152361	-0,106487	-0,223059	-0,151942	0,185612
MeanAV-O	0,586754	0,448078	0,178429	0,060730	0,612430	0,501655	0,049671	0,262982	0,093472	0,298563	-0,083947	0,239700	0,608768	0,467253	0,142417	0,187888	0,284460	0,210289	-0,464439	-0,356632	-0,530701	-0,173454	0,274650
MeanAV-C	0,406544	0,527334	0,144630	0,328691	0,444978	0,613200	0,140837	0,519235	0,194022	0,315737	0,046971	0,473293	0,388417	0,523317	0,143792	0,415087	0,432065	0,269862	-0,269235	-0,279825	-0,576722	-0,038901	0,044913
MEAN-O-FOAM	0,540435	0,458657	0,534032	0,239261	0,512408	0,604039	0,487871	0,582055	0,158435	0,257373	0,340592	0,538381	0,517512	0,454618	0,535424	0,544327	0,258970	0,331124	-0,545672	-0,493028	-0,763981	-0,293792	0,202388
MEAN-C-FOAM	0,171851	0,313187	0,238213	0,385719	0,135882	0,242667	0,283424	0,372095	-0,053718	0,289489	0,141927	0,455279	0,168439	0,310588	0,260489	0,528717	0,042437	0,163800	-0,180219	-0,163525	-0,212099	-0,203361	0,219040
VSS 1	-0,258229	-0,188763	-0,230327	-0,076068	-0,236439	-0,125485	-0,235065	-0,042625	0,019372	-0,247552	-0,203580	-0,112847	-0,240679	-0,187380	-0,222147	-0,122755	0,461520	0,239420	0,181590	0,119879	0,068620	0,014505	-0,152385
DHI 1	-0,227547	-0,235577	-0,168497	0,052432	-0,242043	-0,171172	-0,213790	-0,009117	0,082804	-0,066260	-0,097968	-0,000257	-0,207153	-0,228068	-0,159638	-0,050238	0,265780	0,309718	0,185159	0,151732	0,110275	0,066220	-0,236823
DGI 1	-0,408677	-0,346885	-0,408231	-0,254337	-0,430271	-0,336784	-0,292673	-0,459241	-0,089267	-0,211470	-0,142463	-0,248228	-0,393387	-0,344281	-0,421314	-0,404971	-0,128649	-0,273802	0,623772	0,676171	0,647855	0,550625	-0,602294
BBS 1	-0,483661	-0,281867	-0,449503	-0,158700	-0,488880	-0,337948	-0,324793	-0,460827	-0,166768	-0,152957	-0,192571	-0,239018	-0,503494	-0,291234	-0,473775	-0,369713	-0,267320	-0,331273	0,712869	0,734603	0,756032	0,579404	-0,557192
Tinneti 1	-0,389099	-0,373952	-0,476138	-0,284080	-0,409020	-0,431634	-0,320889	-0,522037	-0,084153	-0,346917	-0,169980	-0,323639	-0,371259	-0,368413	-0,468809	-0,439111	-0,255800	-0,284274	0,548479	0,612430	0,730141	0,398935	-0,372510
FR 1	-0,454020	-0,172048	-0,227428	-0,015115	-0,429970	-0,230985	-0,144033	-0,257813	-0,282418	-0,017845	-0,010032	-0,154017	-0,504913	-0,199110	-0,262327	-0,225148	-0,258940	-0,368604	0,674785	0,600618	0,598998	0,432993	-0,513934
TUG 1	0,256984	0,166013	0,100845	0,091702	0,266900	0,224488	-0,030023	0,381834	0,203080	0,359028	-0,121970	0,274520	0,294600	0,189520	0,110211	0,288750	0,021901	0,284150	-0,461335	-0,458967	-0,462444	-0,366577	0,415733

Color map of p value for correlation coefficient

N=40

p<=	0,001	0,010	0,025	0,050	0,100	0,150	0,200	0,350	0,500	1

Variable	LEN-O 2	LEN-C 2	LEN-O FOAM 2	LEN-C FOAM 2	SURF-O 2	SURF-C 2	SURF-O FOAM 2	SURF-C FOAM 2	MAXAV-O 2	MAXAV-C 2	MAXAV-O FOAM 2	MAXAV-C FOAM 2	MeanAV-O 2	MeanAV-C 2	Mean-O FOAM 2	Mean-C FOAM 2	VSS 2	DHI 2	DGI 2	BBS 2	Tinetti 2	FR 2	TUG 2
LEN-O	0,000	0,000	0,167	0,303	0,000	0,000	0,565	0,021	0,593	0,075	0,973	0,034	0,000	0,000	0,255	0,105	0,076	0,193	0,002	0,012	0,000	0,380	0,160
LEN-C	0,012	0,000	0,336	0,038	0,006	0,000	0,330	0,001	0,247	0,043	0,778	0,003	0,019	0,000	0,345	0,009	0,007	0,133	0,113	0,077	0,000	0,838	0,867
LEN-O FOAM	0,000	0,003	0,000	0,063	0,001	0,000	0,001	0,000	0,394	0,117	0,023	0,000	0,001	0,003	0,000	0,000	0,128	0,047	0,000	0,000	0,000	0,048	0,259
LEN-C-FOAM	0,768	0,362	0,433	0,052	0,717	0,714	0,239	0,369	0,553	0,148	0,619	0,215	0,739	0,385	0,382	0,070	0,687	0,836	0,671	0,709	0,568	0,664	0,375
SURF-O	0,001	0,003	0,427	0,383	0,000	0,000	0,889	0,008	0,325	0,075	0,988	0,058	0,001	0,002	0,537	0,153	0,086	0,214	0,003	0,045	0,000	0,503	0,211
SURF-C	0,061	0,020	0,743	0,101	0,020	0,001	0,895	0,001	0,194	0,151	0,757	0,005	0,070	0,020	0,767	0,027	0,025	0,172	0,067	0,111	0,000	0,826	0,930
SURF-O FOAM	0,004	0,006	0,004	0,181	0,006	0,000	0,005	0,000	0,366	0,136	0,035	0,001	0,007	0,007	0,004	0,002	0,194	0,119	0,005	0,017	0,000	0,245	0,537
SURF-C FOAM	0,494	0,634	0,847	0,348	0,752	0,878	0,900	0,640	0,194	0,155	0,866	0,369	0,476	0,642	0,897	0,286	0,175	0,327	0,888	0,769	0,740	0,448	0,243
MAXAV-O	0,316	0,185	0,567	0,923	0,106	0,047	0,367	0,312	0,639	0,446	0,949	0,290	0,198	0,137	0,532	0,461	0,364	0,237	0,259	0,869	0,220	0,893	0,489
MAXAV-C	0,062	0,012	0,893	0,025	0,045	0,001	0,899	0,001	0,094	0,070	0,844	0,002	0,069	0,013	0,888	0,010	0,010	0,181	0,304	0,291	0,003	0,802	0,851
MAXO-FOAM	0,000	0,001	0,002	0,379	0,000	0,000	0,001	0,002	0,201	0,089	0,102	0,006	0,000	0,001	0,001	0,003	0,059	0,020	0,002	0,007	0,000	0,119	0,255
MAX-C-FOAM	0,354	0,030	0,234	0,013	0,413	0,028	0,172	0,003	0,573	0,019	0,472	0,001	0,337	0,029	0,194	0,001	0,523	0,210	0,348	0,513	0,167	0,349	0,252
MeanAV-O	0,000	0,004	0,271	0,710	0,000	0,001	0,761	0,101	0,566	0,061	0,607	0,136	0,000	0,002	0,381	0,246	0,075	0,193	0,003	0,024	0,000	0,284	0,086
MeanAV-C	0,009	0,000	0,373	0,038	0,004	0,000	0,386	0,001	0,230	0,047	0,773	0,002	0,013	0,001	0,376	0,008	0,005	0,092	0,093	0,080	0,000	0,812	0,783
MEAN-O-FOAM	0,000	0,003	0,000	0,137	0,001	0,000	0,001	0,000	0,329	0,109	0,032	0,000	0,001	0,003	0,000	0,000	0,107	0,037	0,000	0,001	0,000	0,066	0,210
MEAN-C-FOAM	0,289	0,049	0,139	0,014	0,403	0,131	0,076	0,018	0,742	0,070	0,382	0,003	0,299	0,051	0,105	0,000	0,795	0,313	0,266	0,313	0,189	0,208	0,174
VSS 1	0,108	0,243	0,153	0,641	0,142	0,440	0,144	0,794	0,906	0,124	0,208	0,488	0,135	0,247	0,168	0,450	0,003	0,137	0,262	0,461	0,674	0,929	0,348
DHI 1	0,158	0,143	0,299	0,748	0,132	0,291	0,185	0,955	0,611	0,685	0,548	0,999	0,200	0,157	0,325	0,758	0,097	0,052	0,253	0,350	0,498	0,685	0,141
DGI 1	0,009	0,028	0,009	0,113	0,006	0,034	0,067	0,003	0,584	0,190	0,381	0,122	0,012	0,030	0,007	0,010	0,429	0,087	0,000	0,000	0,000	0,000	0,000
BBS 1	0,002	0,078	0,004	0,328	0,001	0,033	0,041	0,003	0,304	0,346	0,234	0,137	0,001	0,068	0,002	0,019	0,095	0,037	0,000	0,000	0,000	0,000	0,000
Tinneti 1	0,013	0,017	0,002	0,076	0,009	0,005	0,044	0,001	0,606	0,028	0,294	0,042	0,018	0,019	0,002	0,005	0,111	0,075	0,000	0,000	0,000	0,011	0,018
FR 1	0,003	0,288	0,158	0,926	0,006	0,152	0,375	0,108	0,077	0,913	0,951	0,343	0,001	0,218	0,102	0,162	0,107	0,019	0,000	0,000	0,000	0,005	0,001
TUG 1	0,109	0,306	0,536	0,574	0,096	0,164	0,854	0,015	0,209	0,023	0,453	0,086	0,065	0,241	0,498	0,071	0,893	0,076	0,003	0,003	0,003	0,020	0,008

Legend:

LEN-O - **Length of trajectory Condition 1;**

LEN-C - Length of trajectory Condition 2;

LEN-O FOAM - Length of trajectory Condition 3;

LEN-C FOAM - Length of trajectory Condition 4;

SURF-O - Surface of COP Condition 1;

SURF-C - Surface of COP Condition 2;

SURF-O FOAM - Surface of COP Condition 3;

SURF-C FOAM - Surface of COP Condition4;

MAXAV-O - Max angular velocity Condition 1;

MAXAV-C - Max angular velocity Condition 2;

MAXAV-O FOAM - Max angular velocity Condition 3;

MAXAV-C FOAM - Max angular velocity Condition 4;

VSS – Vertigo Symptom Scale

DHI – Dizziness Handicap Inventory

DGI – Dynamic Gait Index

BBS - Berg Balance Scale

FR – Functional Reach

TUG – Timed Up and Go test

1 – before VRT

2 – after VRT
